# Length matters: Effects of fishing gear and fishing behavior on the catch efficiency of demersal seines

**DOI:** 10.1016/j.heliyon.2024.e37953

**Published:** 2024-09-17

**Authors:** Thomas Noack, Florian Berg, Lotte Kindt-Larsen

**Affiliations:** a DTU Aqua, Technical University of Denmark, Kemitorvet, 2800, Kgs. Lyngby, Denmark; b Institute of Marine Research, Post Box, 1870 Nordnes, 5817, Bergen, Norway

**Keywords:** Danish seining, Gillnet, MiniSeine, Seal-fishery-conflict, Seal-safe fishing

## Abstract

Raiding seals pose a big problem to gillnet fishers in areas with high seal abundances. As moving to active gears could be a potential solution for that problem, one gear of particular interest in areas with relatively flat sea bed structure is the so-called “MiniSeine” – a demersal seine that is reduced in size so it can be operated from small vessels. Besides its ability to catch most of the species targeted by gillnet fishers, it offers various advantages compared to other active gears. To reduce the gear in size to fit on a small vessel, the present study assessed how seine rope length (4 coils vs 8 coils), seine rope diameter (18 mm vs. 22 mm) and seine net configurations of different sizes and shapes (three different designs) affect the catch efficiency and the ratio of fish below minimum conservation reference size (MCRS). In general, shorter seine ropes (4 coils) resulted in significantly lower catches than longer ones (8 coils), except for the smallest seine configuration. Larger seine net configurations and longer seine ropes caught generally less fish below MCRS, both up to around 10 %. The seine rope diameter did not affect catches significantly. As potential “adjusting screw” to counteract lower catches due to shorter seine ropes, the effect of different layout patterns of the seine ropes on the catch efficiency was assessed. Laying out the second seine rope perpendicular to the first one can increase the fishing area to more than three times while CPUE showed a tendency to be increased compared to the standard layout pattern. Besides its importance for a successful development of the MiniSeine, these findings are of equal interest for the large-scale demersal seine fishery.

## Highlights

• Seine rope length increases CPUE (here kg/h) and decreases < MCRS ratio (amount of undersized fish) of plaice significantly.
• Results indicate positive effect of seine net size on CPUE and <MCRS ratio.
• Seine rope diameter does not affect CPUE or < MCRS ratio.
• Adaption of fishing behavior (set out pattern) can increase CPUE.
• The findings are relevant in designing the MiniSeine, but are important for larger demersal seiners as well.

## 1 Introduction

The Baltic Sea grey seal (*Halichoerus grypus*) population has inceased from 2000 to 4000 individuals in the early 1980s to >38.000 individuals in 2019 [1,2]. This has led to increasing conflicts between seals and passive fisheries [3,4], encouraging the development of seal-safe fishing gears. Seals can either damage the caught fish in a way not allowing it to be sold, or remove the fish entirely – both leading to significant losses of income to the fishery [3]. Besides being seal-safe, alternative gears need to be able to be operated from small vessels by a single fisher (as gillnetters mostly are) and catch the desired target species. Although Atlantic cod (*Gadus morhua*) has belonged to the top target species in the Baltic for a long time, declining cod stocks in the Baltic Sea have led to an action plan (Council Regulation (EC) 2020/1781) restricting cod fisheries. This in turn highlights the need for flexibility, i.e. alternative gears should be able to catch other species than solely cod – e.g. flounder (*Platichthys flesus*) or plaice (*Pleuronectes platessa*) as valuable flatfish species. Fish pots can fulfil the requirement of catching target fish while being seal-safe, but catches depend on factors such as size, shape and soak time [5,6]. Until now, catches have been largely dominated by cod, exhibiting varying catch efficiencies [5,7,8].

Another gear that fulfils the requirements listed above is a downscaled demersal seine – a so-called “MiniSeine” (Fig. 1). Although the use of demersal seines is limited to areas characterized by relatively flat and even grounds, such areas overlap with many of the current gillnetting location and the number of such areas in the Baltic Sea is high. As seal depredation on active gears is considered to be low [9], interactions between seals and the MiniSeine can be expected to be minor. Based on the knowledge from larger commercial vessels, demersal seining is able to efficiently catch various species including species of potential interest for gillnetters like cod and plaice [10]. Additionally, the quality of fish caught in demersal seines is high [11], which can have positive effects on the profit per unit of sold fish. The reason is that that catches spend little time in the gear [12], thus interactions with other biological and non-biological parts of the catch or net parts are limited. The idea of the MiniSeine is to scale down the entire demersal seining system to a level, which is operational by single fishers on small gillnetter vessels. Such reductions in dimension can mainly be adjusted by three parameters: i) seine rope length, ii) seine rope diameter and iii) seine net size and shape.

**Fig. 1:**
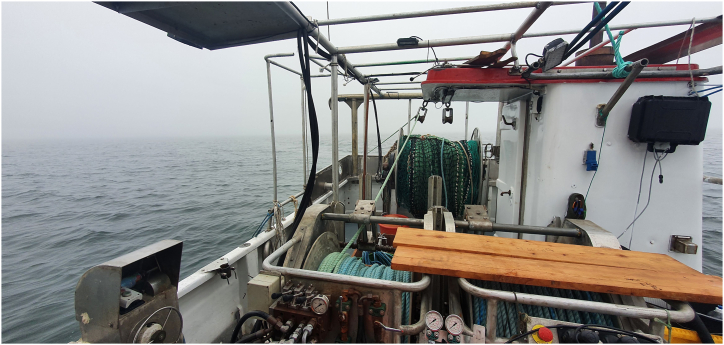
Mini seine system consisting of two rope drums with three coils á 220 m of seine rope (front), net drum (back) and rope-guiding rollers (on top of net drum) rigged on 11 m vessel.

The aim of the present study was to identify the effects the parameters seine rope length, seine rope diameter and seine configuration have on the catch efficiency and further to investigate, if adaptions to the lay-out pattern could compensate for potential catch losses by increasing the swept area. The outcome of the present study provides the basis for any following research on the innovative gear MiniSeine, but is equally valid for the large-scale demersal seining sector.

## 2 Material & methods

### 2.1 Study site and gear specifications

**Fig. 2:**
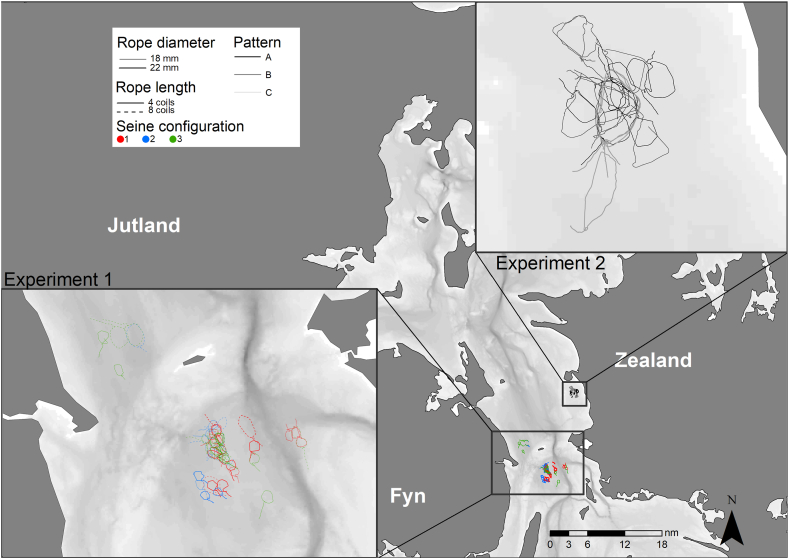
Overview map – fishing operations of both experiments (Experiment 1 “Gear characteristics”, Experiment 2 “Layout patterns”).

All trials were carried out in the Great Belt (ICES Subdivision 27.3.c.22; Fig. 2) between April and August 2020. The first experiment (Experiment 1: “Gear characteristics”) focused on three seine gear-related aspects: i) seine rope length, ii) seine rope diameter and iii) seine net configuration. It was conducted in close proximity to the East Bridge of the Great Belt Fixed Link (Fig. 2, “Experiment 1”), a common plaice fishing ground for gillnetters and the fishing vessel FA50, *Ingrid*, which conducted the trials. FA50 is a comparatively small Danish seiner (overall length 12 m, engine power: 82 kW), which usually fishes with 8 coils (∼220 m each, producing two sets of ∼1760 m each) of seine rope 22 mm in diameter (“Randers Reb”, 0.45 kg·m−1 in air). Each seine rope was separated into two parts of equal length, allowing to fish with either four or eight coils of seine rope, thus testing how seine rope length affects catch efficiency. To evaluate if the seine rope diameter influences catch efficiency, a second set of seine ropes of 18 mm in diameter (“Randers Reb”, 0.34 kg·m−1 in air) was used. To investigate how seine net shape and size affects the catches, three different seine configurations were tested during the experiment (Table 1). All seine nets were equipped with the same kind of tapered codend (4 mm polyethylene double twine; nominal mesh size: 125 mm; 70 open meshes in circumference; length: 3 m; number of selvedges: two with four meshes included in each selvedge).

**Table 1:** Overview – seine types applied in Experiment 1 “Gear characteristics” including information about total length of the individual seine net configurations (including codend), groundgear, meshes around fishing circle (#) and way of vertical spread.

Seine	Length	Groundgear	#	Vertical spread
1	35.5 m	31 m (12 mm “Taifun”) with 106 rubber discs (diameter: 8–12 cm; spacing: 15–37 cm)	267	Bridles (6 m)
2^a^/2	36 m	22 m (12 mm “Taifun”) with 45 rubber discs (diameter: 10 cm; spacing: 50 cm), 11 bobbins (diameter: 17 cm) and 1.7 kg lead-chain	215	Bridles (10 m)
3^a^/3	27 m	21 m (10 mm “Taifun”) with 75 rubber discs (diameter: 7.5–12 cm; spacing: 15–25 cm) and 2 kg lead-chain	129	Dan Leno (0.4 m)

a Groundgear not tightened.

**Fig. 3:**
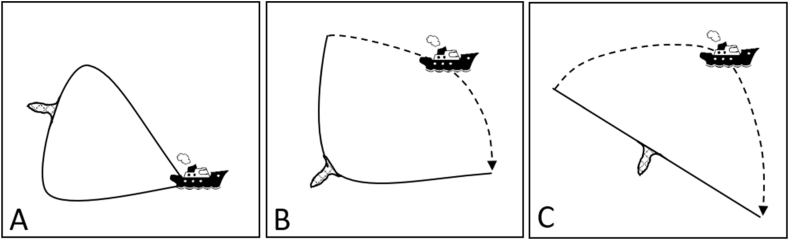
Seine fishing patterns in Experiment 2 “Layout patterns”. Stippled line represents part of fishing track, which is supposed to be towed on the rope.

The second experiment (Experiment 2: “Layout pattern”) compared catches between hauls with three different rope layout patterns (Fig. 3). Layout pattern A refers to the original way of demersal seine fishing. In pattern B, the second seine rope is laid out perpendicular to the first one, while in pattern C, as an extreme case, both ropes are laid out in a straight line (Fig. 3). The trial was conducted in Musholm Bay (Fig. 2, “Experiment 2”) in shallow waters of maximum 10 m as required by the surface connection system (SCS) [12], which allowed tracking specific points of the seine rope during the fishing process. The entire Experiment 2 was conducted with 18 mm seine ropes and seine configuration 2 (Table 1).

### 2.2 Experiment 1: “gear characteristics”

#### 2.2.1 Experimental setup

The period of Experiment 1 was separated into three experimental blocks depending on the gear used (Appendix A1). Each block consisted of two or three fishing trips where each fishing trip consisted of three fishing days (Appendix A1, Appendix A2). Due to COVID-19 restrictions, the majority of the experiment was conducted as a self-sampling study, i.e. data collection took place by the fishers themselves and scientists participated in the cruise only sparsely. In case scientific staff joined, data collection was done by them and more parameters like individual fish lengths was recorded. Fishing took place in a commercial manner. The fishing gear used, however, was changed between the three blocks (Appendix A1, Appendix A2). In block 1, the vessel's own seine rope of 22 mm in diameter was used while the seine configuration was switched between the days. After the first two trips, the fisher pointed out that the footropes of both seine configurations 2^a^ and 3^a^ needed to be tightened in order to fish properly. Therefore, both footropes were tightened, i.e. seine configurations 2^a^ and 3^a^ were replaced by seine configuration 2 and 3 (Appendix A1, Appendix A2, Table 1). As this change was a major modification of the gear, seine configurations 2^a^ and 3^a^ cannot be considered properly rigged and were therefore excluded from any analyses. On each day, two to three hauls using four or eight coils of seine rope, respectively, were conducted. In block 2, it was fished with only four coils of rope – two or three hauls with either 18 mm and two or three hauls with 22 mm seine ropes each day. Again, the seine configuration was changed between the days. Block 3 was designed like block 1, except that only 18 mm seine rope was used.

For each haul, the fishers recorded catch in kg separated by species and by individuals below and above Minimum Conservation Reference Size (MCRS). Additionally, fishing location and depth were recorded and a GPS logger (Renkforce G-PORTER GP-102+; accuracy: 2.5 m circular error probability) recorded the vessel's position every 5 s.

#### 2.2.2 Data analysis

The analyses for Experiment 1 focused only on catches of the target species plaice. A generalized linear mixed model (GLMM) was used to identify potential effects of seine rope length, seine rope diameter and seine configuration on catch efficiency (given as catch per unit effort, CPUE). CPUE was defined as plaice catches in kg per 60 min haul duration. This standardization of CPUE was needed to account for differences in haul duration. Due to the non-normal distribution, CPUE was log-transformed for the modeling approach. A backward selection was applied to find the best explaining model structure, where the initial model included a full interaction between the three categorical variables of interest (rope length, rope diameter, seine configuration) as fixed effects, and trip as a random effect to account for potential differences in conditions between the trips. The final model to explain CPUE was:

$${CPUE}_{h}=\beta_{1}\times {Rope\_coils}_{h}+\beta_{2}\times {Seine\_configuration}_{h}+{\beta_{3}\times {Rope\_coils}_{h}\times {Seine\_configuration}_{h}+b}_{h}+\varepsilon_{h}$$

b_h_ ∼ N(o, σ^2^_b_)

ε_h_ ∼ N(o, σ^2^_ε_)

Where h stands for haul, b for fishing trip as random effect, and ε for the residual error. The same approach has been applied to the ratio of fish below MCRS (<MCRS ratio; catches of plaice < MCRS/all catches of plaice) resulting in the same final model as given above for CPUE. Significant differences among the interaction of seine rope coils and seine configuration on the CPUE and <MCRS ratio were identified using a Tukey's HSD post hoc test. The results of the Tukey's HSD post hoc tests are displayed in the corresponding figures as compact letters where groups that do not share common letters are significantly different (p < 0.05).

All analyses were carried out in R Statistical Software [13], using the “glmmTMB” package [14] to conduct the modelling and the “DHARMa” package [15] to examine model diagnostics.

### 2.3 Experiment 2: “layout pattern”

#### 2.3.1 Experimental setup

In Experiment 2, six SCS were attached to the fishing gear – two on each seine rope and one on each seine net wing. Along with the GPS logger on the fishing vessel, those allowed to track and record movement of fishing vessel, seine ropes and seine net [12]. The net height was recorded by depth loggers (Star-Oddi CTD; depth accuracy: ±0.6 %). This information provided the base for creating simplified haul animations for each of the tested seine rope layout patterns (Fig. 3). Both attaching and removing SCSs required the usual fishing operation to be stopped for approximately 30 s. Due to this stopping, the herding of fish could have negatively affected the catch efficiency, but not the fishing pattern itself. Therefore, additional hauls without SCS were conducted as reference data from “non-affected” fishing hauls. However, the total catch was not significantly influenced by stopping the fishing operation to detach the SCSs (ANOVA, p = 0.16). Thus, hauls with and without SCSs were combined for the following analysis. Similar to Experiment 1, catches (in kg) were recorded separately by species, both below and above MCRS, along with information on fishing location and depth for every haul.

#### 2.3.2 Data analysis

Simplified haul animations were created following the protocol described in Noack et al. [12]. The change of fishing location (Fig. 2) resulted in catches dominated by flounder, which was the target species of Experiment 2. Further, only 19 hauls were conducted for the three fishing patterns in total. The primary focus of Experiment 2 was on the fishing pattern as the sole factor of interest. This allowed us to compare catches applying a simple non-parametric bootstrap approach [16]. By resampling the mean flounder catch per haul with 5000 bootstrap repetitions, the Efron 95 % confidence limits (CIs) were estimated [16]. Non-overlapping CIs would hereby indicate significant differences in the means between the seine rope layout patterns. As the layout-procedures require different periods of time (pattern A: short, pattern B: medium, pattern C: long; see Fig. 3 for pattern visualizations), the duration of the entire fishing processes differed between the individual patterns. To account for this in the analyses, not only catch of flounder per haul, but also catch of flounder per hour (catch_flounder [kg]/haul duration [min]∗60) has been estimated for each haul and compared between the three different patterns. Due to the scarcity of data, the impact of various layout patterns on fish sizes was not analyzed.

## 3 Results

### 3.1 Experiment 1: “gear characteristics”

In total, 78 valid hauls were conducted, which took – depending on the length of seine rope (4 vs 8 coils) – between 37 min and 80 min on average and covered between 0.29 and 1.25 km^2^ on average (Table 2). Catches were generally rather small (13.0–63.9 kg on average) with plaice being the dominant species (Table 2; see Appendix A2 for details about all species and hauls). <MCRS ratio for plaice differed considerably with 15–42 % of the plaice caught being below MCRS on average (Table 2). In general, both catches and <MCRS ratio were characterized by rather high variation (Table 2).

**Table 2:** Overview – fishing operations: Experiment 1 “Gear characteristics”. Given estimates are mean values including min- and max-values in brackets.

Coils	Dia	Seine	Hauls	Haul duration [min]	Covered area [km2]	Catch_Total [kg]	Catch_Plaice [kg]	<MCRS-ratio Plaice [%]
4	18	1	11	39 (35–44)	0.33 (0.29–0.38)	17.9 (7.0–49.5)	13.2 (4.0–43.0)	30.7 (13.0–50.0)
4	18	2	8	39 (36–41)	0.34 (0.30–0.38)	20.6 (5.0–33.8)	15.9 (3.0–28.0)	28.0 (12.3–50.0)
4	18	3	9	37 (28–46)	0.36 (0.24–0.54)	29.0 (5.5–56.0)	23.7 (3.5–49.0)	38.4 (20.0–57.1)
4	22	1	10	41 (39–45)	0.40 (0.32–0.46)	15.5 (6.6–29.0)	11.6 (5.0–26.0)	28.6 (14.3–42.9)
4	22	2	6	40 (33–44)	0.29 (0.26–0.34)	13.0 (6.5–18.2)	9.3 (5.0–15.0)	26.0 (16.7–40.0)
4	22	3	7	37 (35–39)	0.33 (0.31–0.37)	18.0 (11.0–26.0)	13.1 (5.0–22.0)	41.6 (30.8–54.5)
8	18	1	4	73 (71–79)	1.14 (1.14–1.15)	61.3 (39.3–91.3)	48.8 (36.0–68.0)	19.5 (11.8–33.3)
8	18	2	8	78 (40–102)	1.16 (0.39–1.54)	56.0 (22.3–122.0)	31.2 (15.0–60.5)	29.7 (9.9–52.0)
8	18	3	5	80 (67–102)	1.25 (0.84–1.48)	36.7 (17.0–93.0)	27.4 (9.0–78.0)	18.3 (9.1–33.3)
8	22	1	6	77 (68–95)	1.16 (1.16–1.16)	63.9 (48.0–87.1)	50.6 (33.0–67.0)	14.8 (3.5–23.1)
8	22	2	2	76 (75–76)	NA	53.7 (53.0–54.3)	41.5 (40.0–43.0)	21.8 (18.6–25.0)
8	22	3	2	77 (75–78)	NA	21.0 (13.0–29.0)	12.5 (4.0–21.0)	39.3 (28.6–50.0)

**Fig. 4:**
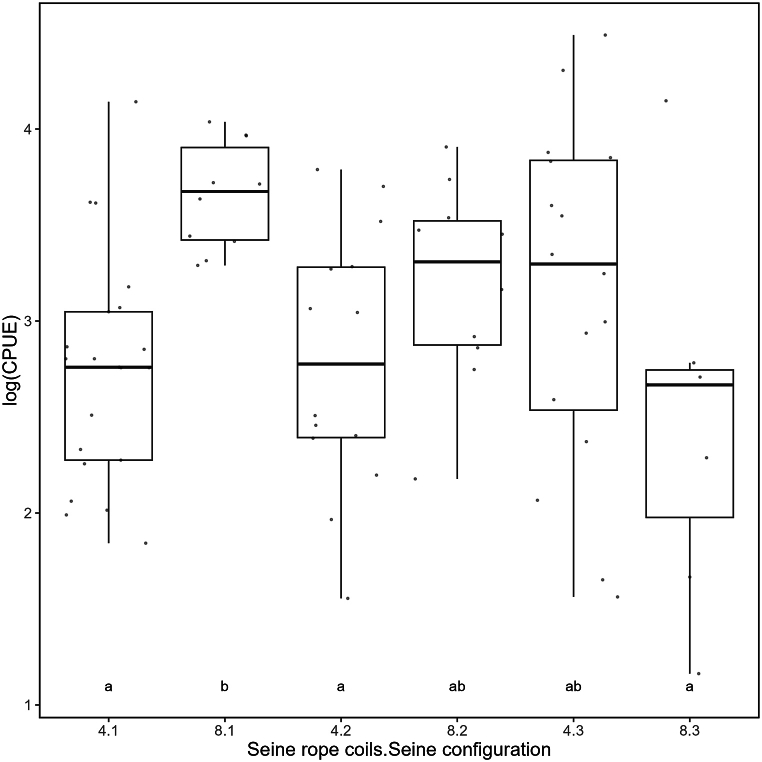
Logarithmic CPUE as catch in kg per hour for plaice in Experiment 1 “Gear characteristics” separated by the different combinations of seine rope coils and seine configuration. Statistical analysis revealed a significant interaction between seine rope coils and configuration, thus, groups that do not share common letters are significantly different (p < 0.05).

Within seine configuration 1 and 2, CPUE of plaice increased significantly when fishing with eight instead of four coils, while for seine configuration 3 the CPUE of plaice was reduced (Tukey's HSD test, p < 0.05, Table 3, Fig. 4). When using four coils of seine rope, seine configuration 1 and 2 resulted in similar CPUEs, but it was slightly higher for configuration 3 (Fig. 4). On the other hand, configuration 1 and 3 resulted in highest and lowest CPUE, respectively, when eight coils of seine rope were used.

**Table 3:** GLMM output for CPUE of plaice (log-transformed) from Experiment 1 “Gear characteristics”.

Predictor	Estimate ±SE	Z	p
Intercept	2.741 ± 0.143	19.158	**<0.001**
Rope_coils			
8	0.910 ± 0.252	3.612	**<0.001**
Seine configuration			
2	0.056 ± 0.226	0.247	0.805
3	0.402 ± 0.218	1.847	0.065
Interactions			
Rope8:Seine2	−0.508 ± 0.370	−1.373	0.170
Rope8:Seine3	−1.563 ± 0.389	−4.014	**<0.001**
Random effect			
Trip	Variance: <0.001	StdDev. <0.001

**Fig. 5:**
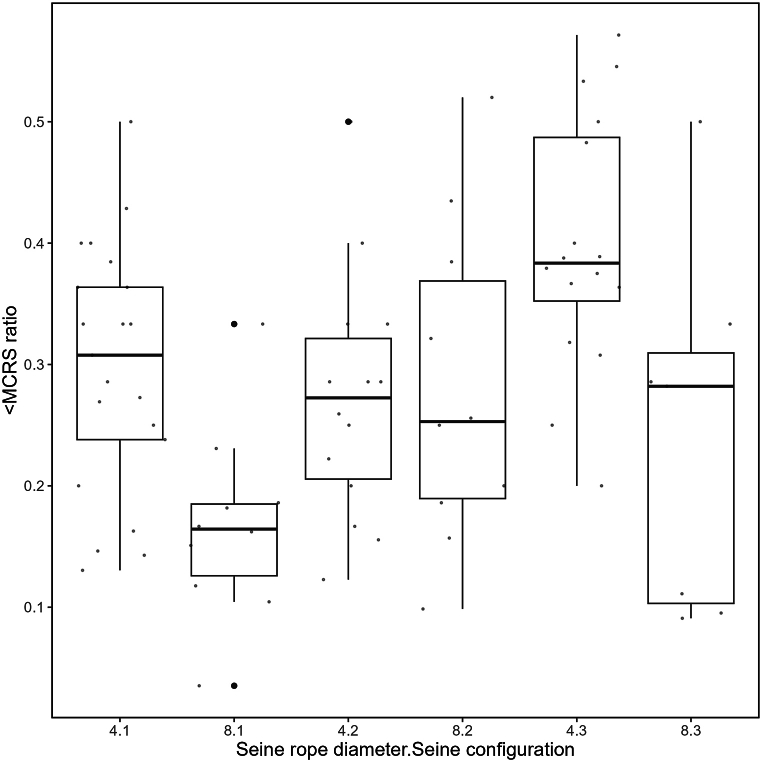
Ratio of plaice below minimum conservation reference size (<MCRS ratio) in Experiment 1 “Gear characteristics” separated by the different combinations of seine rope coils and seine configuration. Statistical analysis revealed a significant interaction between rope coils and configuration, thus, groups that do not share common letters are significantly different (p < 0.05).

<MCRS ratio of plaice was affected significantly by seine configuration (Tukey's HSD test, p < 0.05, Table 4, Fig. 5). Similar to the CPUE, seine configuration 1 and 2 differed from configuration 3. Both caught less fish below MCRS compared to seine configuration 3 when using four coils. In contrast, when using eight coils, seine configuration 1 yielded in the lowest ratio compared with configuration 2 and 3 (Table 4, Fig. 5).

**Table 4:** GLMM output for < MCRS-ratios of plaice from Experiment 1 “Gear characteristics”.

Predictor	Estimate ±SE	Z	p
Intercept	0.295 ± 0.026	11.455	**<0.001**
Rope_coils	−0.131 ± 0.039	−3.37	**<0.001**
8			
Seine configuration			
2	−0.035 ± 0.034	−1.037	0.3
3	0.093 ± 0.033	2.826	**0.005**
Interactions			
Rope8:Seine2	0.151 ± 0.056	2.724	**0.006**
Rope8:Seine3	−0.007 ± 0.059	−0.124	0.902
Random effect			
Trip	Variance: <0.001	StdDev. <0.001

Both the CPUE and the <MCRS ratio were not affected by the seine rope diameter (ANOVA, p > 0.05).

### 3.2 Experiment 2: “layout pattern”

Nineteen valid hauls were conducted within Experiment 2 (Table 5; see Appendix A3 for haul details). For seven hauls, SCSs and depth loggers had been mounted to the seine gear for the performance description of the three different layout patterns A, B and C (see Appendix A4-A6 for respective animations). The reference layout pattern, pattern A (Fig. 3, left), was the simplest one to deploy. Here the entire gear needed to be set out as a kind of circle with the seine net being the middle part of it. The mean fishing area encircled by this method was ∼0.3 km^2^ (Table 5). Layout pattern B (Fig. 3, middle) as a medium extension was also relatively simple to carry out, but required steering the vessel into the direction of the anchor buoy before reaching the end of the second seine rope (see animation in Appendix A5 at timestamp: 00:16:20) in order to ensure maneuverability of the vessel. Else there was a risk that the second seine rope would act like an anchor. Using this method, the mean fishing area could be enlarged to ∼0.5 km^2^ (Table 5). Layout pattern C (Fig. 3, right) tested the extreme case by setting out both seine ropes in a straight line. Similarly as for pattern B and for the same reason, it was very important to change the sailing direction of the vessel before reaching the end of the second seine rope (see animation in Appendix A6 at timestamp: 00:20:40). Laying out the seine ropes after pattern C could expand the mean fishing area covered by the seine ropes to ∼0.9 km^2^ (Table 5).

**Table 5:** Overview – fishing operations: Experiment 2 “Layout patterns”. Given estimates are mean values including min-values and max-values in brackets.

Pattern	Hauls	Covered area [km^2^]	Haul duration [min]	Catch_Total [kg]	Catch_Flounder [kg]
A	9	0.30 (0.24–0.41)	51 (36–81)	21.6 (10.1–37.3)	20.9 (10.1–36.0)
B	6	0.50 (0.45–0.58)	58 (49–70)	34.0 (10.1–86.8)	33.1 (10.1–84.0)
C	4	0.94 (0.69–1.06)	77 (69–84)	29.1 (20.7–33.2)	28.2 (20.2–32.0)

**Fig. 6:**
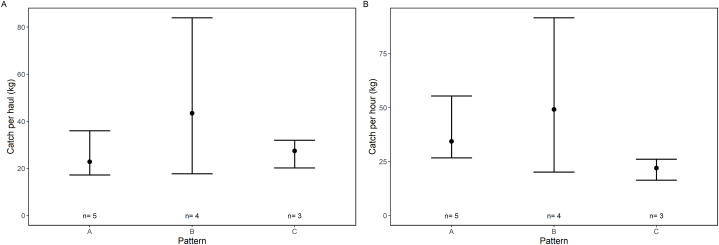
Bootstrapping results (±CI) for catches in kg of flounder per haul (left) and per hour (right) in Experiment 2 “Layout patterns” separated by fishing pattern.

Although the area covered could be increased to more than three times the reference value (pattern A), mean haul duration for pattern C (77 min) was only about 26 min longer than for pattern A (51 min, Table 5). Catches were dominated by flounder with mean total catches per haul of 21.6, 34.0 and 29.1 kg per haul for pattern A, B and C, respectively (Table 5). Pattern B showed the highest range in catches as further demonstrated by wide CIs in the bootstrapped results (Fig. 6). Mean catches of flounder per haul appeared to be rather similar for pattern A and C with B having the potential of increasing this value (Fig. 6A). When translating this information to catch of flounder per hour to account for the different haul durations, the relation between A and B remained similar, but the value for pattern C decreased (Fig. 6B).

## 4 Discussion

The present study investigated how gear characteristic and fishing behavior related aspects may affect catches of a demersal seine. The study is to our knowledge the first study identifying length of the seine ropes, the seine configuration and seine rope layout pattern as important factors determining catches of demersal seines. The results thus serve as important information when assessing a MiniSeine system as potential seal-safe alternative to gillnets, but are equally important for the large-scale demersal seine sector.

### 4.1 Seine rope length

The only gear-related factor that significantly affected CPUE of plaice individually was the seine rope length. This relates to the size of the area, where the seine ropes pass by and herd fish, i.e. the swept area. As longer seine ropes increase the number of fish that can be herded, the obvious recommendation would be: the longer the seine ropes, the better. Although this conclusion would be valid from a catch efficiency point of view, it is not the aim of the present research as the entire system should fit on a small vessel. Therefore, it is again about evaluating how long the seine ropes can be, but still fit onboard the desired vessels. An advantage of shorter seine ropes would be that they could be operated in structured areas, around reefs or close to wrecks. This requires, however, excellent knowledge about local conditions. Furthermore, it highlights the need to investigate how catch efficiency can be improved when fishing with shorter ropes – e.g. by adapting the layout pattern as in Experiment 2 (see. 4.4).

A potential explanation for lower < MCRS ratios (i.e. share of undersized fish in the catch) when fishing with longer ropes is the difference in physiology between smaller and larger fish. As swimming capabilities of larger fish are higher and exhaustion is reached later [17], herding over a longer period is likely to work better for large fish than for small fish. After some time of herding, smaller fish reach a point of fatigue and get overrun by the seine ropes. This means that, contrary to trawling – where fish that are herded in front of the trawl and then stop swimming will enter the trawl [18] – fish that cease swimming while being herded by seine ropes will escape the fishing process. This aspect and the corresponding consequences in terms of bycatch of unwanted small fish need to be borne in mind when thinking about the seine rope length to be used.

### 4.2 Seine configuration

Although individual seine configuration has not been identified as a significant factor in determining CPUE, the combination of eight coils and seine configuration 3 was found to significantly reduce CPUE. This could either be because of the low sampling size of that combination, but it could also indicate some seine rope length-dependent mechanism when fishing with seine configuration 3. However, differences in CPUE between the potential gears seem to not have a strong effect on catch efficiency of plaice. An explanation why plaice catches of the small seine net (seine configuration 3) were not smaller than for the larger ones (seine configurations 1 and 2) could be that fish in front of the net are simply herded further into the middle of the net by the seine ropes when using a net with shorter groundgear, thus still end up in the path of the seine net. This finding is contrary to the existing knowledge for demersal trawls, where increasing the length of the footrope, thus also increasing the opening of the trawl mouth, is a common way to improve catch performance [19]. This highlights the difference in the importance for the herding of the fish between seine ropes for demersal seines and sweeps for trawls. Consequently, the net can be rather small, which facilitates handling, reduces production, maintenance costs, and further reduces space required on deck. However, shortening the net might result in higher numbers of unwanted undersized fish as displayed by a significantly higher < MCRS ratio for seine configuration 3. A likely explanation is that fish, which entered the net, have less chance of escaping through the meshes in front of the codend as the gear parts are shorter, thus time and possibilities for escape attempts are reduced when compared to longer nets. This issue could, however, be tackled by applying suitable measures in the codend (e.g. adapted mesh size). Another negative aspect of smaller nets is that emptying large catches could be demanding depending on available deck structure (crane, etc.) as the catch needs to be divided into smaller shares, which in turn need to be brought on board one after the other.

### 4.3 Seine rope diameter

To save additional space on board, the rope drums could be reduced in size by using thinner rope (e.g. 18 mm as in the present study), thus less rope volume to store. As shown in Experiment 1, neither the catch rates nor the <MCRS-ratio of plaice were affected by the seine rope diameter, indicating that the reduction in the diameter trialed here did not affect their herding performance. Despite not leading to negative effects on catches, thinner seine ropes may cause two other issues. First, they tear faster, i.e. maintenance costs for repairing or replacing worn and broken rope parts might exceed the slightly lower acquisition costs. The magnitude of this effect might, however, be reduced when used in connection with a smaller and lighter seine net as the tensile forces on the ropes would be reduced. Second, thinner seine ropes might dig into the seabed more easily when fishing on muddy grounds, causing the fishing gear to get stuck (personal observation). Although releasing the ropes is possible, the haul and the herded fish are lost in most cases. It is therefore a matter of finding a good balance. After experience from these and previous trials with 15 mm seine ropes, we can conclude that seine ropes with a diameter of 15 mm tear quite fast and dig frequently into the sediment. On the other hand, seine ropes of 18 mm diameter are more robust and are less prone to get stuck, but they are still thinner than those typically used in the seine fishery [12,20], thus fit smaller drums.

### 4.4 Layout pattern

Counteracting the fact that shorter ropes mean less catches was the aim of Experiment 2. As this part of the study was restricted to water depths <10 m, it might be speculated that the gear performs differently or that herding of the fish works differently when fishing in deeper waters. According to fishers, however, fishing procedures are independent of depth, which indicates that fishers assume gear geometry patterns and herding capabilities of the seine ropes to be similar.

The two alternative layout patterns B and C were both able to extend the covered area while keeping the increase in mean haul duration low. Both required accurate and careful setting of the seine rope when approaching the end of the second rope. The vessel needed to be turned in the desired direction beforehand as the rope would else act like an anchor and affect the vessel's maneuverability negatively. Although pattern C could cover about three times as much seabed as pattern A by not even doubling the mean haul duration, the catch efficiency was not increased. A likely explanation for this phenomenon is that the herding performance of the seine ropes was affected when fishing with pattern C. It might have affected the angle of attack of the seine ropes [21] in a negative way so the swimming ability of the fish allowed them to escape after being herded or not to be herded at all. That the swimming ability of fish is an essential part of the capture process in active gears has been shown for roundfish [22] as well as flatfish [23]. Linked to the angle of attack is the speed the seine ropes are moving with over the seabed. As the animation for pattern C shows (Appendix 6), it is only the second seine rope, which is moving in the beginning of the retrieval process. In contrast to the first seine rope, which is laying still, it is moving relatively fast over the seabed. It might be the case that the speed even exceeds the speed the targeted fish can swim with, i.e. even though the fish would try to escape from the approaching seine rope, the rope moves faster than the fish can swim and rolls over them. As explained in “4.1 Seine rope length”, this could particularly be the case for smaller fish as the swimming capacities are less, but should be investigated further. Future studies could use anchored cameras in the fishing area to record the herding of fish. Such cameras would further allow an assessment of the impacts of demersal seines on the seabed. This topic has been considered from a more theoretical point of view before [24,25], but statements about the gear's advantages including its low impacts on benthos [26] are often still based on assumptions. Although their effects are considered to be lower than for other demersal mobile gears [24,25], ropes of demersal seine can have effects on protruding organisms like macroalgae or sea pens (Pennatulacea) and remove them. Such interactions can happen between both seine net as well as seine ropes. A potential tool to be used is the observation platform described in Noack et al. [12].

Although the number of hauls in this experiment is low in general, the results for layout Pattern B showed some trends, indicating its potential for increasing catches. A likely explanation is that pattern B represents a compromise of pattern A and pattern C, i.e. the fishing area is extended while the herding performance of the seine ropes is not affected. Furthermore, pattern B was the favorite of the skipper during both experiments (personal communication) although he usually applies pattern A when fishing commercially. For future fishing trips, he is planning to use pattern B more often as it seems to increase catches. A similar procedure called “towing on the rope” is usually conducted by larger Danish anchor seiners [12]. Instead of laying both seine ropes out in a symmetrical pattern, the end of the second seine rope is reached in good distance to the buoy and towed back to it. For future research, more catch comparison hauls for the different patterns could be suggested in order to strengthen the data collected.

A drawback of the entire study is the generally rather small catches. Repeating the trials is therefore advisable although catch patterns are expected to remain the same. A surprising aspect of the study in relation to catches is the relatively high MCRS ratio for plaice, i.e. that a rather large meshed codend (125 mm nominal mesh size) retained numerous small fish. One potential explanation is the occurrence of sea weed in the fishing area during the trials, which might have blocked the meshes and prevented small fish from escaping as suggested by Wileman et al. [27].

## 5 Conclusion

Refocusing on the final aim of this research – the potential transition of gillnet fishers to demersal seining – vessel size is an important aspect to consider. Although the trials have been conducted on a small commercial Danish seiner of 12 m, the outcomes can be transferred to the MiniSeine as potential seal-safe alternative gear to gillnets as well as to other scales of demersal seining without any adjustments, i.e. the results might also prove useful for the large-scale demersal seine fishery: i) Longer seine ropes increase catch efficiency and decrease the amount of small plaice. ii) The results indicate similar tendencies for the size of the seine net. iii) Contrary, seine rope diameter does not affect CPUE nor < MCRS ratio. iv) Proper rigging of the gear is essential to ensure efficient fishing. v) Adaption of fishing behavior (set out pattern) can increase CPUE.

A general outcome of the study seems to be that compromises need to be accepted when transferring such gear designed and refined for larger vessels to smaller ones. Experiments on an 11 m gillnetter with a MiniSeine system (Fig. 1; properly designed for such vessels), proved the principles of the system to also work under such conditions (unpublished data). Both, the present study (drastic differences in catch efficiency depending on if the groundgear was tightened or not) as well as the previous trials with the gillnetter in general showed further that proper education of fishers not having experience with that kind of gear is essential for achieving successful fishing. The trials with the MiniSeine system are planned to continue on other vessels and in other areas to further proof the ability of it to work under various conditions. Within these experiments, catch comparison trials between the MiniSeine system and gillnets should be considered as those will allow to compare catch efficiencies of both gears. Such information is inevitable when talking about the viability of a new fishing gear and when answering the question, if the MiniSeine can serve as seal-safe alternative for gillnets.

## CRediT authorship contribution statement

**Thomas Noack:** Writing – review & editing, Writing – original draft, Visualization, Software, Resources, Project administration, Methodology, Investigation, Formal analysis, Data curation, Conceptualization. **Florian Berg:** Writing – review & editing, Software, Methodology, Formal analysis. **Lotte Kindt-Larsen:** Writing – review & editing, Writing – original draft, Validation, Methodology, Conceptualization.

## Declaration of competing interest

The authors declare that they have no known competing financial interests or personal relationships that could have appeared to influence the work reported in this paper.

## Figures and Tables

**figs1:**
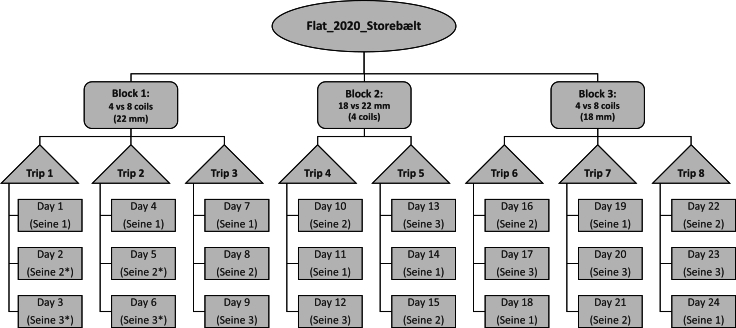


## References

[1] HELCOM (2020).

[2] Varjopuro R. (2011). Co-existence of seals and fisheries? Adaptation of a coastal fishery for recovery of the Baltic grey seal. Mar. Pol..

[3] Königson S., Lunneryd S.G., Stridh H., Sundqvist F. (2009). Grey seal predation in cod gillnet fisheries in the central Baltic sea. J. Northwest Atl. Fish. Sci..

[4] Olsen M.T., Galatius A., Härkönen T. (2018). The history and effects of seal-fishery conflicts in Denmark. Mar. Ecol. Prog. Ser..

[5] Kindt-Larsen L., Berg C.W., Hedgärde M., Königson S. (2023). Avoiding grey seal depredation in the Baltic Sea while increasing catch rates of cod. Fish. Res..

[6] Stavenow J., Ljungberg P., Kindt-Larsen L., Lunneryd S.G., Königson S. (2020). What attracts Baltic sea grey seals to seal-safe cod pots and when do they attempt to attack fish in the pots?. J. Ocean Technol..

[7] Bryhn A.C., Königson S.J., Lunneryd S.-G., Bergenius M.A.J. (2014). Green lamps as visual stimuli affect the catch efficiency of floating cod (*Gadus morhua*) pots in the Baltic Sea. Fish. Res..

[8] Königson S.J., Fredriksson R.E., Lunneryd S.-G., Strömberg P., Bergström U.M. (2015). Cod pots in a Baltic fishery: are they efficient and what affects their efficiency?. ICES J. Mar. Sci. J. Cons..

[9] Cronin M., Gerritsen H., Reid D., Jessopp M. (2016). Spatial overlap of grey seals and fisheries in Irish waters, some new insights using telemetry technology and VMS. PLoS One.

[10] Noack T., Frandsen R.P., Wieland K., Krag L.A., Berg F., Madsen N. (2017). Fishing profiles of Danish seiners and bottom trawlers in relation to current EU management regulations. Fish. Manag. Ecol..

[11] Dreyer B.M., Nøstvold B.H., Midling K.Ø., Hermansen Ø., Lovatelli A., Holthus P.F. (2008). Capture-based Aquaculture.

[12] Noack T., Stepputtis D., Madsen N., Wieland K., Haase S., Krag L.A. (2019). Gear performance and catch process of a commercial Danish anchor seine. Fish. Res..

[13] R Core Team (2023).

[14] Brooks M.E., Kristensen K., Van Benthem K.J., Magnusson A., Berg C.W., Nielsen A., Skaug H.J., Mächler M., Bolker B.M. (2017). glmmTMB balances speed and flexibility among packages for zero-inflated generalized linear mixed modeling. Rom. Jahrb..

[15] Hartig F. (2021). DHARMa: residual diagnostics for hierarchical (multi-level/mixed) regression models. https://cran.r-project.org/web/packages/DHARMa/index.html.

[16] Efron B. (1982).

[17] Nguyen V.Y., Bayse S.M., Einarsson H.A., Ingólfsson Ó.A. (2023). Inferring fish behaviour at the trawl mouth from escape location. PeerJ.

[18] Winger P.D., Eayrs S., Glass C.W., He P. (2010). Behavior of Marine Fishes.

[19] Brinkhof J., Larsen R.B., Herrmann B., Grimaldo E. (2017). Improving catch efficiency by changing ground gear design: case study of Northeast Atlantic cod (*Gadus morhua*) in the Barents Sea bottom trawl fishery. Fish. Res..

[20] Noack T., Frandsen R.P., Krag L.A., Mieske B., Madsen N. (2017). Codend selectivity in a commercial Danish anchor seine. Fish. Res..

[21] O'Neill F.G., Noack T. (2021). The geometry and dynamics of Danish anchor seine ropes on the seabed. ICES J. Mar. Sci..

[22] Breen M., Dyson J., O'Neill F.G., Jones E., Haigh M. (2004). Swimming endurance of haddock (*Melanogrammus aeglefinus* L.) at prolonged and sustained swimming speeds, and its role in their capture by towed fishing gears. ICES J. Mar. Sci..

[23] Ryer C.H. (2008). A review of flatfish behavior relative to trawls. Fish. Res..

[24] Eigaard O.R., Bastardie F., Breen M., Dinesen G.E., Hintzen N.T., Laffargue P., Mortensen L.O., Nielsen J.R., Nilsson H.C., O'Neill F.G., Polet H., Reid D.G., Sala A., Sköld M., Smith C., Sørensen T.K., Tully O., Zengin M., Rijnsdorp A.D. (2016). Estimating seabed pressure from demersal trawls, seines, and dredges based on gear design and dimensions. ICES J. Mar. Sci..

[25] Eigaard O.R., Bastardie F., Breen M., Dinesen G.E., Hintzen N.T., Laffargue P., Mortensen L.O., Nielsen J.R., Nilsson H.C., O'Neill F.G., Polet H., Reid D.G., Sala A., Sköld M., Smith C., Sørensen T.K., Tully O., Zengin M., Rijnsdorp A.D. (2016). Estimating seabed pressure from demersal trawls, seines, and dredges based on gear design and dimensions. ICES J. Mar. Sci..

[26] Suuronen P., Chopin F., Glass C., Løkkeborg S., Matsushita Y., Queirolo D., Rihan D. (2012). Low impact and fuel efficient fishing-Looking beyond the horizon. Fish. Res..

[27] Wileman D., Ferro R.S.T., Fonteyne R., Millar R.B. (1996). Manual of methods of measuring the selectivity of towed fishing gears. ICES (Int. Counc. Explor. Sea) Coop. Res. Rep..

