# Supplementary material for: Length matters: Effects of fishing gear and fishing behavior on the catch efficiency of demersal seines
Source: Heliyon. 2024 Sep 17;10(18):e37953. doi: 10.1016/j.heliyon.2024.e37953 (PMC11447324; doi:10.1016/j.heliyon.2024.e37953)
Supplement: Multimedia component 1 [file mmc1.docx]

Appendix

A1. Experimental design

A2. Raw haul data (incl. catches) from experiment 1 (gear characteristics).

see folder “appendix”

A3. Raw haul data (incl. catches) from experiment 2 (layout patterns).

see folder “appendix”

A4. Animation for pattern A (Haul 14) applied in Experiment 2 “Layout patterns”.

<https://www.dropbox.com/s/ca6989z4bagpqhv/14_A%20%280.40%20km2%29.mp4?dl=0>

A5. Animation for pattern B (Haul 13) applied in Experiment 2 “Layout patterns”.

<https://www.dropbox.com/s/bcxvptjamqw3dky/13_B%20%280.52%20km2%29.mp4?dl=0>

A6. Animation for pattern C (Haul 12) applied in Experiment 2 “Layout patterns”.

<https://www.dropbox.com/s/wkhdv6zqt9kue4f/12_C%20%281.0%20km2%29.avi?dl=0>
